# Supplementary material for: Resolving the full spectrum of human genome variation using Linked-Reads
Source: Genome Res. 2019 Apr;29(4):635–45. doi: 10.1101/gr.234443.118 (PMC6442396; doi:10.1101/gr.234443.118)
Supplement: Supplemental Material [file supp_gr.234443.118_Supplemental_longranger-2.2.zip › longranger-2.2.tar.bz2!/lr2.2-public/lib/stan/cmdstan/stan/doc/api/html/globals_vars.html]

Stan: File Members


|  |  |
| --- | --- |
|  | Stan  2.5.0  probability, sampling & optimization |


- Main Page
- Related Pages
- Namespaces
- Classes
- Files

- File List
- File Members

- All
- Functions
- Variables
- Macros

- \_
- a
- b
- c
- d
- e
- h
- i
- l
- m
- n
- s
- v
- x


All Classes Namespaces Files Functions Variables Typedefs Enumerations Enumerator Friends Macros Pages

### - \_ -

- \_adjARef
  : determinant.hpp
  , log\_determinant.hpp
  , log\_determinant\_spd.hpp
- \_alloc
  : log\_determinant\_spd.hpp
  , mdivide\_left\_ldlt.hpp
  , mdivide\_left\_spd.hpp
- \_alloc\_ldlt
  : log\_determinant\_ldlt.hpp
  , mdivide\_left\_ldlt.hpp
- \_cols
  : determinant.hpp
  , log\_determinant.hpp
  , log\_determinant\_spd.hpp
- \_impl
  : quad\_form.hpp
  , trace\_gen\_quad\_form.hpp
  , trace\_inv\_quad\_form\_ldlt.hpp
  , trace\_quad\_form.hpp
- \_invA
  : log\_determinant\_spd.hpp
- \_ldlt
  : trace\_inv\_quad\_form\_ldlt.hpp
- \_ldltP
  : mdivide\_left\_ldlt.hpp
- \_llt
  : mdivide\_left\_spd.hpp
- \_rows
  : determinant.hpp
  , log\_determinant.hpp
  , log\_determinant\_spd.hpp
- \_sym
  : quad\_form.hpp
- \_value
  : trace\_inv\_quad\_form\_ldlt.hpp
- \_variB
  : trace\_inv\_quad\_form\_ldlt.hpp
- \_variD
  : trace\_inv\_quad\_form\_ldlt.hpp
- \_variRefA
  : mdivide\_left.hpp
  , mdivide\_left\_spd.hpp
  , mdivide\_left\_tri.hpp
- \_variRefB
  : mdivide\_left.hpp
  , mdivide\_left\_ldlt.hpp
  , mdivide\_left\_spd.hpp
  , mdivide\_left\_tri.hpp
- \_variRefC
  : mdivide\_left\_ldlt.hpp
  , mdivide\_left.hpp
  , mdivide\_left\_tri.hpp
  , mdivide\_left\_spd.hpp

### - a -

- A\_
  : determinant.hpp
  , log\_determinant.hpp
  , quad\_form.hpp
  , mdivide\_left.hpp
  , mdivide\_left\_tri.hpp
  , trace\_gen\_quad\_form.hpp
  , trace\_quad\_form.hpp
- add\_var\_f
  : var\_decls\_grammar\_def.hpp
- AinvB\_
  : trace\_inv\_quad\_form\_ldlt.hpp
- alpha\_
  : Eigen\_NumTraits.hpp
  , log\_softmax.hpp
  , softmax.hpp

### - b -

- B\_
  : quad\_form.hpp
  , trace\_gen\_quad\_form.hpp
  , trace\_quad\_form.hpp

### - c -

- C\_
  : mdivide\_left.hpp
  , mdivide\_left\_ldlt.hpp
  , trace\_inv\_quad\_form\_ldlt.hpp
  , mdivide\_left\_spd.hpp
  , quad\_form.hpp
  , mdivide\_left\_tri.hpp
- column\_
  : json\_parser.hpp
- copy\_square\_cholesky\_dimension\_if\_necessary\_f
  : var\_decls\_grammar\_def.hpp

### - d -

- D\_
  : trace\_gen\_quad\_form.hpp
  , trace\_inv\_quad\_form\_ldlt.hpp
- dotval\_
  : Eigen\_NumTraits.hpp
- Dtype\_
  : trace\_inv\_quad\_form\_ldlt.hpp

### - e -

- empty\_range\_f
  : var\_decls\_grammar\_def.hpp
- exp\_val\_
  : log10.hpp

### - h -

- h\_
  : json\_parser.hpp

### - i -

- idx\_
  : log\_softmax.hpp
  , softmax.hpp
- in\_
  : json\_parser.hpp

### - l -

- length\_
  : dot\_product.hpp
  , Eigen\_NumTraits.hpp
  , squared\_distance.hpp
  , sum.hpp
- line\_
  : json\_parser.hpp

### - m -

- M\_
  : mdivide\_left.hpp
  , mdivide\_left\_ldlt.hpp
  , mdivide\_left\_spd.hpp
  , mdivide\_left\_tri.hpp

### - n -

- N\_
  : mdivide\_left.hpp
  , mdivide\_left\_ldlt.hpp
  , mdivide\_left\_spd.hpp
  , mdivide\_left\_tri.hpp
- next\_char\_
  : json\_parser.hpp

### - s -

- set\_double\_range\_lower\_f
  : var\_decls\_grammar\_def.hpp
- set\_double\_range\_upper\_f
  : var\_decls\_grammar\_def.hpp
- set\_int\_range\_lower\_f
  : var\_decls\_grammar\_def.hpp
- set\_int\_range\_upper\_f
  : var\_decls\_grammar\_def.hpp
- size\_
  : log\_softmax.hpp
  , softmax.hpp
  , dot\_self.hpp
- softmax\_alpha\_
  : log\_softmax.hpp
  , softmax.hpp

### - v -

- v1\_
  : dot\_product.hpp
  , Eigen\_NumTraits.hpp
  , squared\_distance.hpp
- v2\_
  : dot\_product.hpp
  , Eigen\_NumTraits.hpp
  , squared\_distance.hpp
- v\_
  : dot\_self.hpp
  , sum.hpp
- validate\_decl\_constraints\_f
  : var\_decls\_grammar\_def.hpp
- validate\_double\_expr\_f
  : var\_decls\_grammar\_def.hpp
- validate\_identifier\_f
  : var\_decls\_grammar\_def.hpp
- validate\_int\_data\_expr\_f
  : var\_decls\_grammar\_def.hpp
- validate\_int\_expr\_f
  : var\_decls\_grammar\_def.hpp

### - x -

- xs\_
  : sort\_indices.hpp


---

[ Stan Home Page ]

*© 2011–2014,
Stan Development Team.*
